# Integration of single-cell transcriptome and chromatin accessibility and its application on tumor investigation

**DOI:** 10.1093/lifemedi/lnae015

**Published:** 2024-04-26

**Authors:** Chunyuan Yang, Yan Jin, Yuxin Yin

**Affiliations:** Institute of Systems Biomedicine, Department of Pathology, School of Basic Medical Sciences Peking University, Peking-Tsinghua Center for Life Sciences, Peking University Health Science Center, Beijing 100191, China; Institute of Systems Biomedicine, Department of Pathology, School of Basic Medical Sciences Peking University, Peking-Tsinghua Center for Life Sciences, Peking University Health Science Center, Beijing 100191, China; Institute of Systems Biomedicine, Department of Pathology, School of Basic Medical Sciences Peking University, Peking-Tsinghua Center for Life Sciences, Peking University Health Science Center, Beijing 100191, China; Institute of Precision Medicine, Peking University Shenzhen Hospital, Shenzhen 518036, China

**Keywords:** single-cell transcriptome sequencing (scRNA-seq), single-cell chromatin accessibility sequencing, tumor microenvironment, single-cell multi-omics, assay for transposase-accessible chromatin

## Abstract

The advent of single-cell sequencing techniques has not only revolutionized the investigation of biological processes but also significantly contributed to unraveling cellular heterogeneity at unprecedented levels. Among the various methods, single-cell transcriptome sequencing stands out as the best established, and has been employed in exploring many physiological and pathological activities. The recently developed single-cell epigenetic sequencing techniques, especially chromatin accessibility sequencing, have further deepened our understanding of gene regulatory networks. In this review, we summarize the recent breakthroughs in single-cell transcriptome and chromatin accessibility sequencing methodologies. Additionally, we describe current bioinformatic strategies to integrate data obtained through these single-cell sequencing methods and highlight the application of this analysis strategy on a deeper understanding of tumorigenesis and tumor progression. Finally, we also discuss the challenges and anticipated developments in this field.

## Introduction

Gene expression serves as the foundation of most cellular activities, which is comprised of transcription, translation, and then, in many cases, post-translational modifications. Transcription is tightly regulated by the activity of transcription factors (TFs). The genomic DNA is tightly packed with histones to form nucleosomes, and in a quiescent state, is inaccessible to TFs [1]. Therefore, genome accessibility emerges as one of the most crucial indicators for gene regulatory networks (GRN). The establishment of sequencing technologies detecting chromatin accessibility has transformed our comprehension of cellular activities, which includes chromatin immunoprecipitation followed by sequencing (CHIP-seq) [2], deoxyribonuclease I (DNase I)-hypersensitive site sequencing (DNase-seq) [3], assay for transposase-accessible chromatin with sequencing (ATAC-seq) [4], and formaldehyde-assisted isolation of regulatory elements sequencing (FAIRE-seq) [5]. However, these methods primarily rely on bulk tissues, limiting their application to cellular heterogeneity investigations. Consequently, there arises a compelling need for the employment of single-cell sequencing techniques in the analyses of complex tissues.

Cancer ranks as one of the most lethal diseases worldwide, and improvements in tumor treatments are limited primarily due to the great cellular heterogeneity within bulk tumors. In recent decades, the application of single-cell RNA sequencing (scRNA-seq) has broadened the understanding of both tumor cells and tumor microenvironments (TMEs) [6]. This knowledge has revolutionized tumor treatment strategies by providing novel potential treatment targets. Nevertheless, intricate mechanisms underlying these transcriptomic alterations and TME dysfunctions remain incompletely understood.

Here, we review the advancements of scRNA-seq and scATAC-seq, provide an overview of current data integration methods, and elucidate the advantages of employing this single-cell multi-omics strategy on tumor investigations. Finally, we discuss present challenges and future perspectives for the application of single-cell multi-omics in cancer research.

## Progress in single-cell transcriptomic sequencing

**Figure 1.**
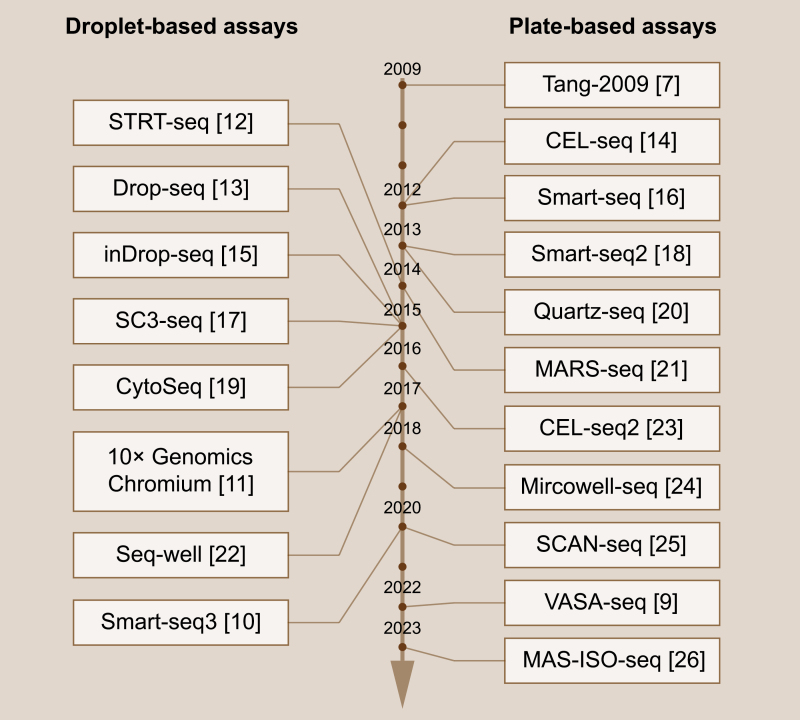
**Time line of single-cell RNA sequencing methods.**Representative droplet-based (left) and plate-based (right) scRNA-seq techniques are listed from top to bottom according to their invention time, with corresponding references listed behind.

Since the introduction of scRNA-seq method by Tang et al. in 2009 [7], this sequencing technology has rapidly garnered global attention. Based on different cell division strategies, scRNA-seq methods can be classified into plate-based assays and droplet-based assays [8]. In plate-based assays, cells are divided into micro-wells using flow cytometry, followed by RNA extraction and DNA library construction within the same micro-well. This methodology allows the sequencing of full-length transcripts, which helps to distinguish RNA at allele and isoform resolution. Over the past decade, the application of plate-based scRNA-seq techniques has expanded from coding transcripts to non-coding transcripts, including long non-coding, short non-coding, and non-polyadenylated protein-coding transcripts [9]. However, limited by the flow cytometry cell separation strategy, the throughput of plate-based scRNA-seq is relatively restricted. On the other hand, droplet-based assays employ a microfluid system to simultaneously separate and label cells with unique identifiers, facilitating high-throughput sequencing. The recently developed Smart-seq3 balanced the long-read sequencing and high-throughput sequencing requirements, enabling large-scale characterization of cell types and states [10]. Notably, the Chromium platform by 10× genomics [11] has been dedicated to increasing sequencing throughput, currently allowing the simultaneous sequencing of up to one million cells collected from up to 128 specimens in a single run. This remarkable high-throughput method has significantly accelerated the discovery of rare cell populations. Considering the distinct advantages of plate-based assays and droplet-based assays, both techniques have found wide applications in the past decade (Fig. 1), leading to enormous advancements in tumor biology.

Importantly, scRNA-seq stands as a pivotal tool for the identification of significant yet rare cell populations that are seldom detected through bulk sequencing methods. Examples of such cells include cancer stem cells (CSC) [27] and treatment-resistant tumor cells [28]. In colorectal carcinoma (CRC), LGR5^+^ tumor cells have been proven to be the major CSC population, while metastatic sites are predominantly initiated by LGR5^−^ cells [29, 30]. The transformation dynamics of these two populations in diseases remain elusive. Vasquez et al. have uncovered the dynamic states between Lgr5^+^ ve crypt base columnar cells with Lgr5^−^ ve regenerative stem cells, and depicted different ratios of these cell subpopulations in malignant and nonmalignant diseases [31]. In gastric cancer, scRNA-seq has identified multiple subtypes of CSCs, including CD44V6^−^CD133^+^CD166^+^ cells, CD44V6^+^CD133^+^CD166^+^ cells, and their upstream cell populations, namely TROP2^+^CD133^+^CD166^+^ cells [32]. Furthermore, a subpopulation of quiescent stem-like cells plays an important part in chemoresistance and poor outcomes of acute myeloid leukemia revealed by scRNA-seq. Drug-resistant signaling pathways have also been identified in CRC [33]. Cells showing activated oxidative phosphorylation and ATP metabolic states predict chemoresistance potential [34]. Furthermore, scRNA-seq has greatly advanced our understanding of TME components, encompassing cancer-associated fibroblasts (CAFs), endothelial cells [35], and immune cells [36]. During the tumorigenesis process, fibroblasts interact with tumor cells and were trained into CAFs. Through scRNA-seq, CAF heterogeneity in various types of solid tumors has been identified, including pancreatic adenocarcinoma (PDAC) [37, 38], breast cancer [39, 40], lung cancer [41], liver cancer [42], and so on. CAF subpopulations, including myofibroblastic CAFs, inflammatory CAFs, and antigen-presenting CAFs, regulate extracellular matrix remodeling, immune cell recruitment, immune cell activation regulation, and interactions with tumor cells [43]. As for endothelial cells, scRNA-seq has revealed critical metabolic remodeling characteristics during tumorigenesis [44]. Tumor-specific tip-like endothelial cells participant in angiogenesis and tumor immune escape, thereby promoting tumor progression across multiple types of tumor [45]. Furthermore, the understanding of immune cell subpopulations has also greatly benefitted from scRNA-seq. Subclusters of T cells [46], macrophages [47], dendritic cells [48, 49], B cells [50, 51], and neutrophils [52] have been identified. The discovery of tumor-specific immune statuses not only unravels TME heterogeneity but also provides substantial new targets for tumor therapies.

## Progress in single-cell chromatin accessibility sequencing

To fully depict GRN and trace back for transcriptional regulatory mechanisms, various epigenetic analysis methods have been developed. Over the past decade, sequencing techniques evaluating DNA methylation, histone modification, and chromatin accessibility are emerging. As an important gene regulatory mechanism, DNA methylation at CpG island detection using bisulfite conversion followed by next-generation sequencing profiles DNA methylome, whereas the low throughput and high cost limited the wide application of this technique [53, 54]. Histone modification sequencing techniques, including CHIP-seq [55], and subsequent cleavage under targets and tagmentation (CUT&Tag) [56], assess DNA-protein interaction to evaluate gene regulatory mechanisms. However, the intricate protocols and high costs associated with these methods increase the difficulty of broad utilization. In contrast, chromatin accessibility sequencing detects *cis*-regulatory element activity and TF binding sites, thereby unraveling the gene expression regulatory network. Among the various techniques, ATAC-seq has gained widespread use due to its ease of operation, high throughput, and relatively low cost [4]. Hence, we focus on chromatin accessibility detection, especially using ATAC-seq, to unveil gene regulatory mechanisms.

The process of chromatin accessibility detection is composed of two steps: assigning TFs to open chromatin regions, namely peaks in many methods, and subsequent assignment of these open chromatin regions to specific genes. Based on these two processes, many bulk chromatin accessibility methods have been developed [2–5]. However, given the cellular complexity of most tissues, it is not surprising that rare cell types are frequently neglected using these bulk methods. Novel methods that can detect cellular chromatin accessibility at single-cell level are urgently needed. As scRNA-seq techniques began to emerge, the detection of chromatin accessibility began to extend from bulk methods to single-cell methods. Single-cell chromatin accessibility techniques, such as scATAC-seq, facilitate the discovery of multiple cellular states regulated by different TFs and introduce the concept of “regulome” into the assessment of cellular variation [57]. Cell-type-specific chromatin accessibility features have also been identified [58].

The application of these single-cell chromatin accessibility methods has provided novel insights into various physiological and pathological processes [59–64]. In tumors, single-cell chromatin accessibility sequencing has been applied to the investigation of *cis*-elements in both tumor cells and TME components. During lung cancer tumor progression, chromatin accessibility modules transform from normal to metastasis [65]. Additionally, chromatin accessibility sequencing unravels epigenetic regulators in both tumor cell and tumor-infiltrating immune cell of basal cell carcinoma. A comparison of T-cell status before and after programmed cell death protein 1 blockade identifies critical chromatin accessibility regulators during T-cell exhaustion [66]. In terms of tumor treatment, chimeric antigen receptor T cells (CAR-T) therapy has shown promising therapeutic efficacy in various tumor types but may be hindered by T-cell differentiation after injection. However, single-cell chromatin accessibility sequencing helps to improve treatment efficacies. Using scATAC-seq, Jiang et al. identified chromatin accessibility alterations in differentiated T cells, initiated by BATF and IRF4. Knockdown of BATF and IRF4 inhibited T-cell exhaustion and prolonged the validity of CAR-T therapy [67]. In conclusion, chromatin accessibility sequencing provides novel insights into tumor characteristics, facilitates patient prognosis evaluation, and aids in assessing treatment effectiveness [65–67], thereby indicating a critical dimension for tumor investigation.

**Figure 2.**
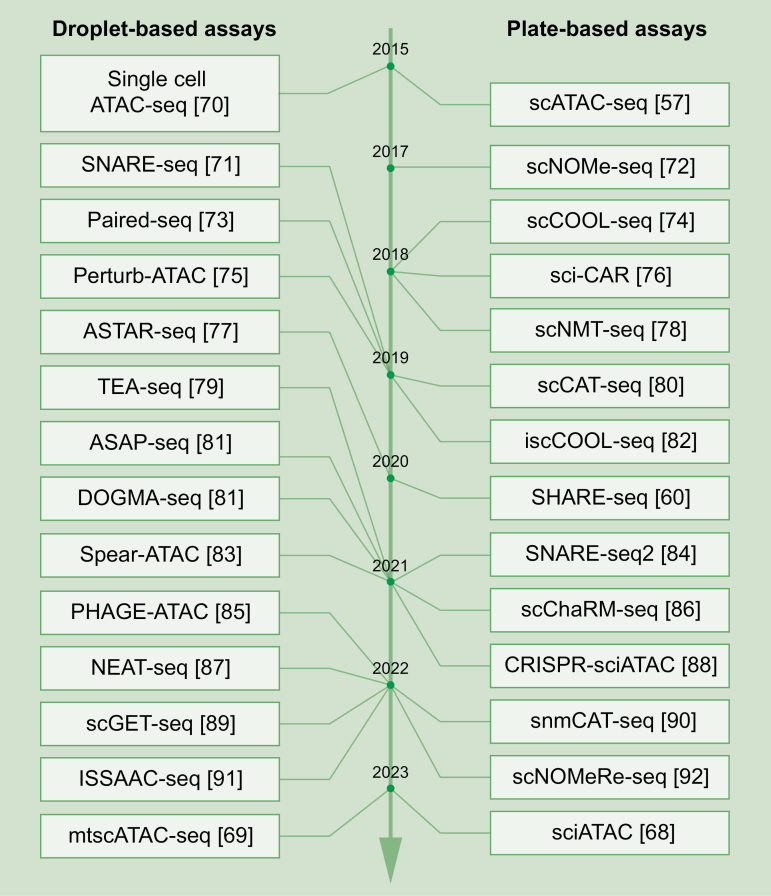
**Time line of single-cell chromatin accessibility sequencing methods.**Representative droplet-based (left) and plate-based (right) single-cell chromatin accessibility sequencing techniques are listed from top to bottom according to their invention time, with corresponding references listed behind.

Over the past decade, the throughput of scATAC-seq techniques has increased from hundreds [57] to hundreds of thousands (> 10^5^ cells) [68], greatly accelerating the discovery of chromatin accessibility during cell-to-cell transitions and prompting the identification of rare cell states. Furthermore, considering the critical functions of mitochondria in cell biology, the investigation of DNA accessibility detection has expanded from genome DNA to mitochondrial DNA [69], introducing a new dimension for GRN investigations (Fig. 2). Notably, to comprehensively depict a whole GRN, a series of multi-omics methods have emerged, including the combinatorial detection of single-cell chromatin accessibility with single-cell transcriptome, single-cell DNA methylation, single-cell genome sequencing, and/or single-cell proteome (Table 1). The comprehensive use of these sequencing techniques not only provides a full landscape of GRN but also helps to identify critical transcriptomic regulators.

**Table 1.** Single-cell multi-omics based on chromatin accessibility sequencing methods

Number of omics	Type of omics	Method	Ref.
Two omics data integration	Chromatin accessibility + DNA methylation	scNOMe-seq	[72]
		scCOOL-seq	[74]
		iscCOOL-seq	[82]
	Chromatin accessibility + transcriptome	sci-CAR	[76]
		scCAT-seq	[80]
		SNARE-seq	[71]
		Paired-seq	[73]
		SHARE-seq	[60]
		ASTAR-seq	[77]
		SNARE-seq2	[84]
		ISSAAC-seq	[91]
		sciATAC	[68]
	Chromatin accessibility + proteome	Perturb-ATAC	[75]
		CRISPR-sciATAC	[88]
		ASAP-seq	[81]
		Spear-ATAC	[83]
		PHAGE-ATAC	[85]
	Chromatin accessibility + genome	scGET-seq	[89]
		mtscATAC-seq	[69]
Three omics data integration	Chromatin accessibility + DNA methylation + Transcriptome	scNMT-seq	[78]
		scChaRM-seq	[86]
		snmCAT-seq	[90]
		scNOMeRe-seq	[92]
	Chromatin accessibility + transcriptome + proteome	TEA-seq	[79]
		DOGMA-seq	[81]
		NEAT-seq	[87]

## Integration of single-cell transcriptome and chromatin accessibility data

Given the distinct types of data obtained for single-cell transcriptome versus chromatin accessibility sequencing, integration of these data types remains a challenging task. From the perspective of cell sources, a comprehensive analysis of scRNA-seq and single-cell chromatin accessibility data can be categorized into either paired sequencing and unpaired sequencing [93]. Paired sequencing data comes from simultaneous detection of both transcriptome and chromatin accessibility, as exemplified by 10× genomics chromium single-cell multiome ATAC + gene expression products [94, 95]. Multifunctional scATAC-seq analysis packages, especially ArchR [96] and signac [97], offer robust chromatin accessibility analyses as well as a direct projection between transcriptome and GRN. The use of paired data analysis tools minimizes the risk of neglecting GRNs in rare cell populations.

**Figure 3.**
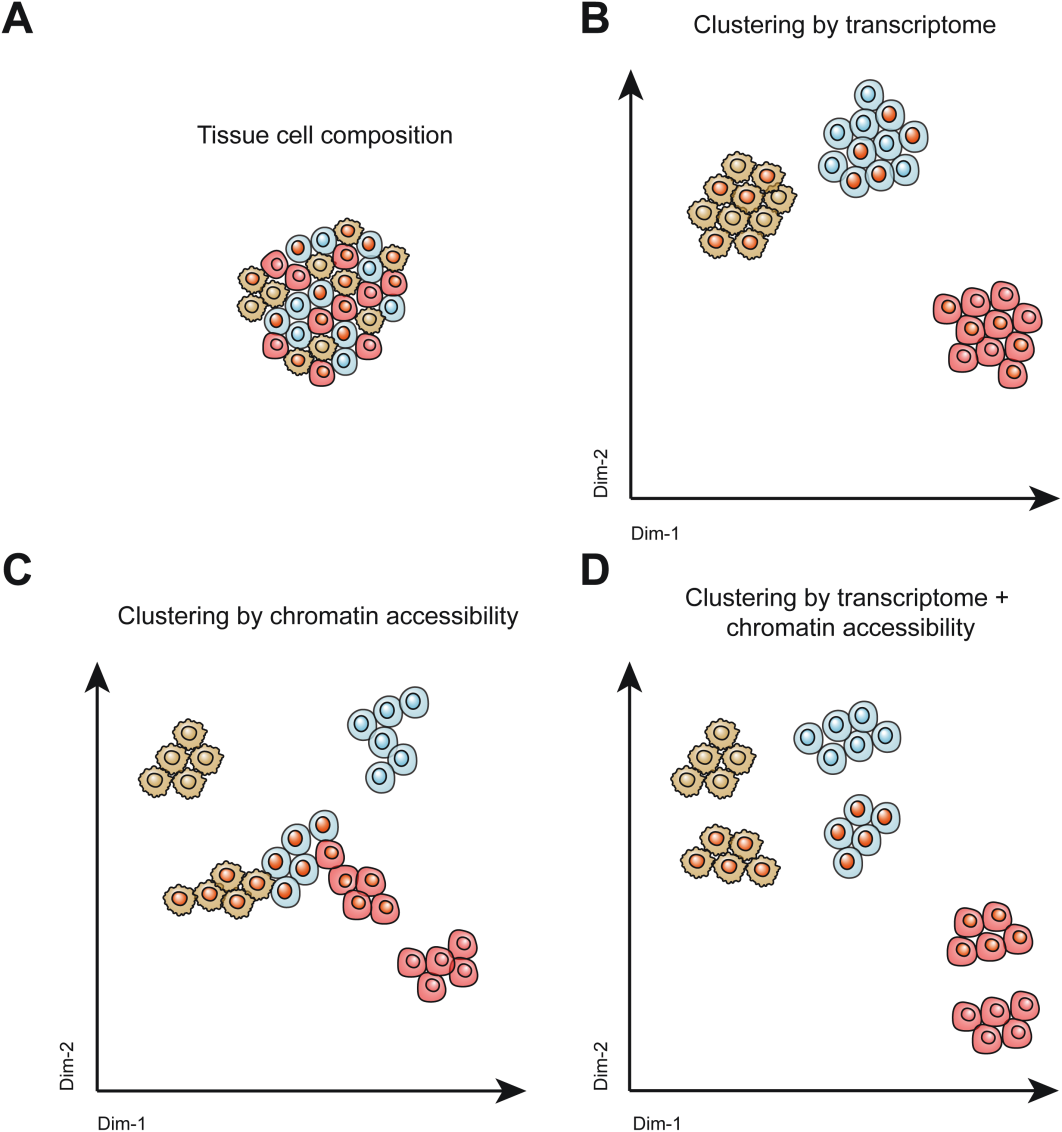
**Cell clustering by different single-cell sequencing techniques.**Shown are representative illustrations of (A) the complex cellular composition within tissues, (B) cell clustering using scRNA-seq methods, (C) cell clustering by single-cell chromatin accessibility sequencing methods, and (D) cell clustering by combining single-cell transcriptome and single-cell chromatin accessibility methods.

However, from a data mining perspective, there is an expectation to match single-cell transcriptome data and single-cell chromatin accessibility data from unpaired cells, such as from those originating from different sequencing experiments of the same organ. Numerous challenges arise in integrating transcriptomic and chromatin accessibility data from unpaired cells. First, the inherent heterogeneities among samples. Individual heterogeneity potentially brings great differences in cell type (or cell state) composition even within the same organ, underlying the fundamental cause of the data integration dilemma. Second, sequencing batch effects. The application of different sequencing platforms, distinct sample preparation strategies, and variations in sequencing depth all influence the matching of cells in transcriptomics and chromatin accessibility data. To overcome these challenges, various mathematical methods have been introduced to align these two types of data, including manifold alignment (AIscEA [98]), variational autoencoders (GLUE [99]), probabilistic modeling (MultiVI [100]), and more (Table 2). The combination of single-cell transcriptome data and single-cell chromatin accessibility data leverages the accurate cell type annotation of scRNA-seq and GRN investigation of single-cell chromatin accessibility sequencing (Fig. 3). Altogether, this comprehensive sequencing strategy facilitate the discovery of GRNs in biological and pathological processes, including developments [101–103], immune reactions [104, 105], chemotherapy responses [106], and tumorigenesis, which will be detailed in the following section.

**Table 2.** Representative methods for integrating scRNA-seq and scATAC-seq data

Tool	Method	Data type	Language	Ref.
AIscEA	Manifold alignment	Unpaired	Python	[98]
MMD-MA	Manifold alignment	Unpaired	Python	[118]
Pamona	Manifold alignment	Unpaired	Python	[119]
SCOT	Gromov-Wasserstein optimal transport	Unpaired	Python	[120]
UnionCom	Manifold alignment	Unpaired	Python	[121]
sc-compReg	Correlation-based	Unpaired	R	[109]
Seurat v3	Correlation-based	Unpaired	R	[122]
scMTNI	Probabilistic graphical model-based	Unpaired	C++	[123]
SOMatic	Toroid topology-based	Unpaired	C++/R	[103]
Dictys	Stochastic process model	Unpaired/paired	Python	[101]
GLUE	Variational autoencoders	Unpaired/paired	Python	[99]
Inferelator 3.0	Regularized regression	Unpaired/paired	Python	[124]
MultiVI	Probabilistic modeling	Unpaired/paired	Python	[100]
ArchR	Corresponding barcode	Paired	R	[96]
DeepMAPS	Autoencoder-like neural networks	Paired	Python	[125]
DIRECT-NET	Constrained optimal cell mapping	Paired	R	[126]
FigR	Constrained optimal cell mapping	Paired	R	[127]
GRaNIE	Pearson’s correlation	Paired	R	[104]
MAGICAL	Hierarchical Bayesian framework	Paired	R	[105]
Pando	Minimum-cost maximum-flow bipartite matching	Paired	R	[102]
scAI	Matrix factorization	Paired	R	[106]
SCENIC+	Unsupervised identification model	Paired	R	[128]
signac	Corresponding barcode	Paired	R	[97]

## Application of single-cell transcriptome and chromatin accessibility assays on tumor investigations

The heterogeneity of tumor cells and the complexity of the TME are considered the leading causes of therapeutic failure. Hence, deciphering the development of tumors at single-cell level is the foundation of precision medicine. In the past few years, researchers using scRNA-seq methods have made substantial inroads into a better understanding of TME heterogeneity, by enabling the compilation of atlases of the TME of many different tumor types [107]. Nevertheless, mechanisms underlying gene regulatory networks are frequently neglected. Recently, analysis of a combination of single-cell transcriptome and chromatin accessibility information has improved our understanding of tumor cells and the TME and may be summarized as follows (Table 3).

**Table 3.** Application of single-cell transcriptome and chromatin accessibility methods on tumor investigations

Cancer type	Sample source	Main findings	Ref.
Acute leukemia	Malignant and normal blood cells	Identification of key TFs (RUNX1, FOS, and NFE2)	[108]
Bladder cancer	Primary and recurrent tumor tissue	Identification of transcription regulator (EZH2) and key TF (TCF7)	[129]
Breast cancer	Primary and metastatic tumor tissue	Identification of chromatin accessible regions in CXCL14-high cancer cells and immune cells	[130]
Chronic lymphocytic leukemia	Malignant and normal blood cells	Identification of a new B cell subtype featured by TOX2 regulatory function	[109]
Clear cell renal carcinoma	Tumor and adjacent tissues	Identification of key TFs (HOXC5, VENTX, ISL1, and OTP)	[111]
Clear cell renal carcinoma	Different tumor subtypes	Identification of distinct chromatin accessibility features in different cancer subtypes	[131]
Colorectal cancer	Primary and metastatic tumor tissue	Chromatin accessibility shifts upon tumor metastasis, and identification of key TF (HNF4A)	[113]
Colorectal cancer	Different stages of tumor	Chromatin accessibility shifts during tumor development, and identification of key TF (RUNX1)	[110]
Colorectal cancer	Pre and postchemotherapy tumor tissue	Chromatin accessibility changes upon drug treatment regulating metabolic states	[132]
Glioma	Different tumor subtypes	Chromatin accessibility difference (CCCTC binding site deletion) according to differences in ATRX expression status	[114]
Male breast cancer	Different types of tumor	Identification of differences in chromatin accessibility of MYC *cis*-regulatory elements in male and female breast cancer	[133]
Multiple myeloma	Subtypes of tumor	Tumor evolution tree mapping according to chromatin accessibility and transcriptome	[134]
Multiple myeloma	Postchemotherapy tumor cells	Chromatin accessibility features in venetoclax-resistant multiple myeloma	[135]
Ovarian and endometrial tumor	Different tumor subtypes	Identification of greater heterogeneity in chromatin accessibility relative to transcriptome	[112]
Multiple (skin, breast, and pancreatic cancer)	Different tumor types	Identification of a cancer-associated fibroblast subtype according to chromatin accessibility	[115]
Retinoblastoma	Tumor at different stages	Tumor evolution trajectory, chromatin accessibility identification of RB-positive tumor-initiating cells	[136]

The employment of single-cell transcriptome and chromatin accessibility strategy has enabled a more comprehensive exploration of the mechanisms of tumorigenesis. For example, compared with normal cells, malignant acute leukemic cells express higher levels of select TFs including RUNX1, FOS, and NFE2 [108]. In addition, in chronic lymphocytic leukemia, a new subtype of B cell has been identified through a comprehensive analysis of scRNA-seq and scATAC-seq, which is featured by the high expression levels of TOX2 [109]. Moreover, as polyps progress to colorectal cancer, the proportion of stemness cells gradually increases, and pre-CAFs as well as regulatory T cells also show evolution patterns. Chromatin accessibility of the RUNX1 gene in CAF and HNF4A gene in epithelial cells suggests the underlying mechanisms of CRC development [110]. Comprehensive analysis of scRNA-seq and scATAC-seq data has unraveled unique chromatin accessibility features of TME in clear cell renal cell carcinoma and identified critical prognostic-indicating TFs [111]. Intriguingly, compared with tumor heterogeneity at single-cell transcriptome level, single-cell chromatin accessibility-level heterogeneity has been found to be greater in multiple myeloma, implying a greater sensitivity of epigenomic methods for detecting tumors [112].

Integrative use of single-cell transcriptomic and chromatin accessibility sequencing in describing tumor progression has also identified many important regulators. Liver metastases of colorectal carcinoma have been found to resemble liver cancer instead of CRCs at both transcriptomic and epigenetic levels. The chromatin accessibility of HNF4A is significantly elevated in liver metastasis, suggesting an important role for HNF4A in colorectal carcinoma progression [113]. Epigenetic remodeling of ATRX-deletion glioma has been identified to be absent of CCCTC-binding sites, thereby showing treatment resistance [114]. In another example, treatment effectiveness has been shown to be influenced by CAFs. Using both scRNA-seq and scATAC-seq, three subtypes of cancer-associated fibroblasts were identified, and the dynamics among these fibroblast subtypes have been shown to correlate with the prognosis of several cancers, including skin cancer, breast cancer, and pancreatic cancer [115]. Importantly, pan-cancer investigation using both snATAC-seq and scRNA-seq has revealed the cancer-specific differentially accessible chromatin regions (DACRs) during cancer evolution, these DACRs primarily locate in *cis*-regulatory regions, including 53% in enhancers and 37% in promoters. Cancer-type specific TFs have also been revealed, including KLF6 and PPARG in PDAC. Furthermore, compared with primary tumors, metastatic tumors show unique TFs. TWIST1 and PBX3 show higher accessibilities during CRC metastasis. GNA13 shows higher accessibility during melanoma metastasis [116].

**Figure 4.**
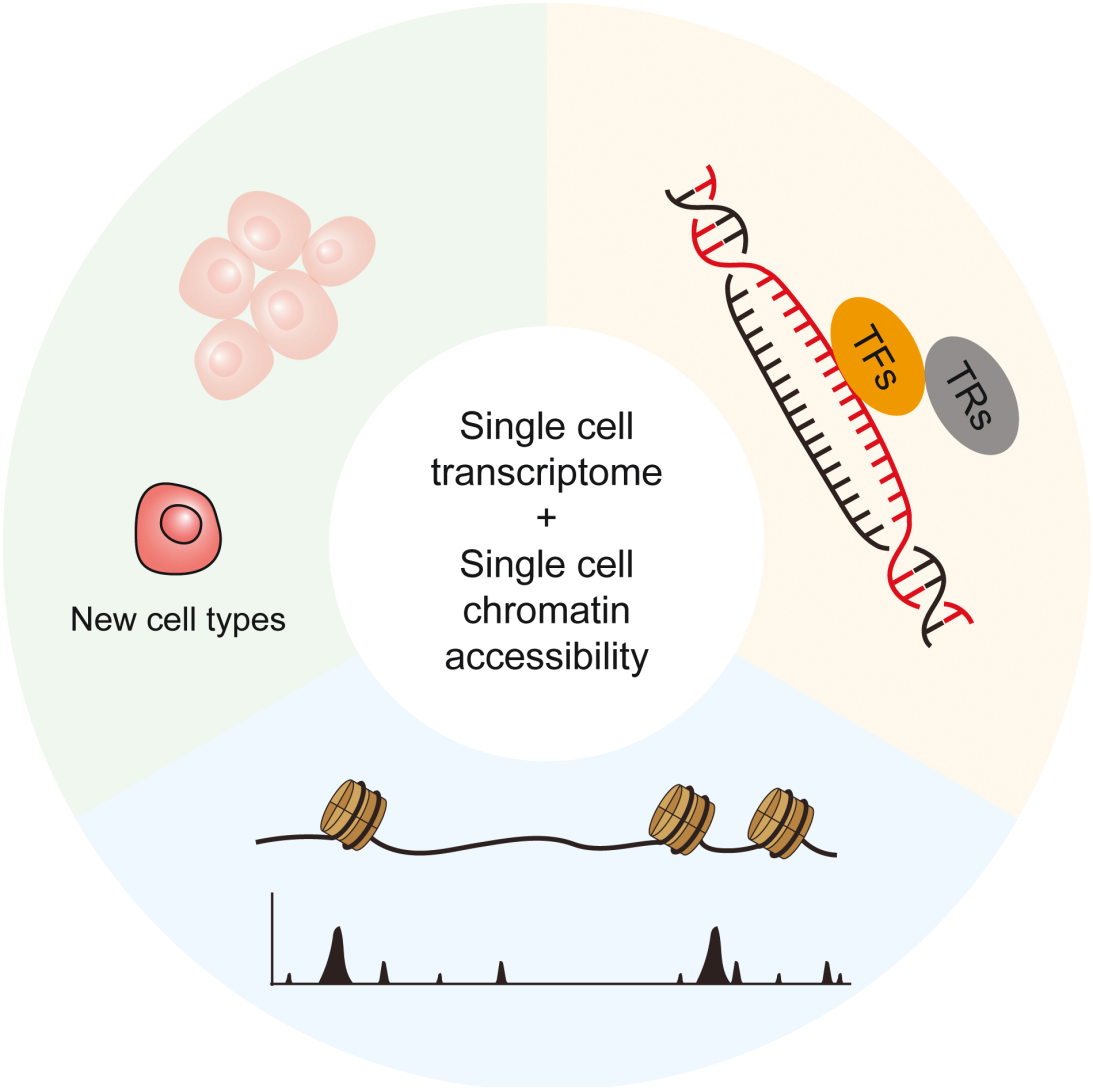
**Graphical abstract.**Schematic graph showing the application of single-cell transcriptome sequencing and single-cell chromatin accessibility sequencing in tumor investigation, which prompts the identification of tumor-specific chromatin accessible regions, transcription factors (TFs), transcription regulators (TRs), and new cell types.

Taken together, these examples demonstrate that the combination of single-cell transcriptome and chromatin accessibility sequencing uncovers the tumor-specific chromatin accessibility regions, TFs, transcription regulators, and novel cell types [117], revolutionizing the study of tumor biology (Fig. 4). However, considering the vast heterogeneity of tumor samples, epigenetic characteristics should be observed in a large number of patients from around the world. The etiology of tumors can dramatically differ among patients from different countries. For example, while hepatitis virus infection is a predominant cause of liver cancer in Asia, alcohol and nonalcoholic steatohepatitis prevail as major causes in Western countries. Previous single-cell transcriptomic sequencing results have shown consistent gene expression characteristics in liver cancer worldwide, regardless of the underlying causes [35]. Nevertheless, investigations into the GRNs of tumors from diverse regions remain unexplored. Therefore, future endeavors should encompass a broader spectrum of districts to comprehensively elucidate tumorigenesis mechanisms.

## Conclusions

Developments in single-cell transcriptomic sequencing and chromatin accessibility sequencing techniques, coupled with the evolutions of data integration tools, have paved the way for GRN investigation in biological and pathological processes, particularly in tumors. This approach has led to the identification of novel cell subpopulations, cell type-specific TFs, transcription regulators, and chromatin regions associated with pathological conditions, all playing critical roles in tumorigenesis. Altogether, the comprehensive assessment of single-cell transcriptome and chromatin accessibility characteristics enhances our understanding of tumors both in baseline conditions and under treatments.

Despite the numerous achievements in sequencing and data analysis, several challenges persist. From the point of transcriptomic or chromatin accessibility sequencing, the presence of alternative splicing and multiple transcription variants complicates the accurate assessment of cell states. Failure to distinguish between transcription variants may result in inaccurate interpretations of sequencing data. From the point of data integration, despite the availability of various tools, the choice of analysis methods can introduce biases to results. The recently developed data integration tools primarily handle two types of input data: trajectories or groups. The former is more suitable for continuous data analysis and has been employed for develop assessments. In contrast, the latter is more suitable for discrete data analysis and applies to the investigation of pathological conditions, including tumorigenesis. Given that tumorigenesis and tumor developments are progressive pathologic processes, accurate tool selection at different steps is paramount. More importantly, from the point of application, although there have been various single-cell multi-omics techniques, high cost, and intricate experimental protocols limit the widespread of many well-performed techniques. To overcome these challenges, researchers must familiarize themselves with the developments and mechanisms of sequencing techniques as well as data processing methods. Only by deep understandings of both experiments and bioinformatic analyses can the researchers choose adequate approaches to address their scientific inquiries. Furthermore, improving the level of industry commercialization is essential to provide more options for the majority of researchers. Finally, with the advances in single-cell multi-omics sequencing and analysis methods, we anticipate deeper insights into tumorigenesis mechanisms and tumor treatment options.
